# Gynecomastia: An ADR due to drug interaction

**DOI:** 10.4103/0253-7613.59929

**Published:** 2009-12

**Authors:** Umme Aiman, M.A. Haseeen, S.Z. Rahman

**Affiliations:** Department of Pharmacology, Jawaharlal Nehru Medical College, AMU, Aligarh, India; 1 Department of Cardio-Vascular and Thoracic Surgery, Safdarjang Hospital, New Delhi, India

**Keywords:** Adverse drug reactions, digoxin, furosemide

## Abstract

Gynecomastia results from conditions that cause an imbalance of estrogenic and androgenic effects on the breast, resulting in an increased or unopposed estrogen action on breast tissue. Approximately 4 to 10% cases of gynecomastia are due to drugs. Both Digoxin and Furosemide are also reported to cause the same condition. Although, chances of gynecomastia could be more if these two drugs are coadministered, but no case report of this adverse effect is ever reported when both are prescribed concurrently. Here we report a case of gynecomastia suspected to have resulted from the coadministation of both the drugs. Probability of the adverse effect due to drug interaction was evaluated by DIPS, which suggests that the adverse drug reaction (ADR) due to DI is “Possible.”

## Introduction

Gynecomastia is defined as a benign enlargement of the male mammary glands, resulting in palpable subalveolar tissue.[1] Gynecomastia results from conditions that cause an imbalance of estrogenic and androgenic effects on the breast, resulting in an increased or unopposed estrogen action on the breast tissue. Approximately 4–10% cases of gynecomastia are due to drugs.[2] Mechanisms by which drugs cause gynecomastia include inhibition of androgen synthesis and/or metabolism (ketoconazole), antagonism at androgen receptor (flutamide, finastride), direct action at estrogen receptors by estrogen-like drugs (estrogen vaginal cream, clomiphine) and displacement of estrogen from sex hormone-binding globulin (thereby increasing the free estrogen level, e.g. spironolactone). Some drugs can directly damage the testis and may cause gynecomastia (busulfan, vincristine).

Digoxin is a cardiac glycoside used for the treatment of congestive heart failure due to its positive inotropic effect by inhibiting Na^+^K^+^ ATPase enzyme on myocardial cells. In addition, it is also used in patients with atrial fibrillation to control the ventricular rate. The adverse effects of digoxin are both cardiac and extracardiac. Gynecomastia is one of the well-known extracardiac side-effects. Furosemide, a loop diuretic, also indicated in congestive heart failure, is also reported to cause gynecomastia.[3] Hence, chances of gynecomastia could be enhanced if these two drugs are coadministered. To our knowledge, there is no report of this adverse effect when both are prescribed concurrently. Here, we report a case of gynecomastia suspected to have resulted from the coadministation of digoxin and furosemide.

## Case History

On February 2008, a 23-year-old male patient attended the surgical out patient department with complaints of dyspnea on exertion for the last 1 year. Dyspnea was progressive in nature and sometimes associated with palpitations. On examination, a diastolic murmur was present in the mitral area and the pulse was irregular. After an electrocardiogram and echocardiography, he was diagnosed as a case of rheumatic heart disease along with mitral stenosis, mild tricuspid regurgitation and atrial fibrillation. He was advised furosemide 40 mg/day and digoxin 0.25 mg/day. At the 3-month follow-up visit, he complained of a lump in his left breast. On examination, a firm, painless lump of 5 cm size was present in the subalveolar region of his left breast whereas the right breast was found to be normal. There was no evidence of any other lump in the body or of testicular enlargement. The hemogram, liver function tests and renal function tests were within normal limits. As the symptom of dyspnea worsened, a decision was made for surgical intervention. The patient underwent mitral valve replacement and, postoperatively, amiodarone was substituted for digoxin. At the same time, furosemide was also stopped. On follow-up visits, it was observed that the lump decreased in size and was barely detectable at 4 months after discontinuation of the suspected drugs.

### Analysis of the case report

The Drug Interaction Probability Scale is designed to assess the probability of a causal relationship between a potential drug interaction and an event.[4] As per the criteria, the score of our case report is in the range of 2–3, which suggests that the adverse drug reaction (ADR) due to DI is “Possible.”

## Discussion

The conditions causing gynecomastia can be physiological and pathological. Physiological causes include neonatal (due to transplacental transfer of maternal estrogens), puberty (high estrogen to androgen ratio in early stages of puberty) and ageing (increased adipose tissue and increased aromatase activity). Pathological causes include hypogonadism (e.g., Klinefelter syndrome), tumors (testicular, ovarian feminizing tumors), liver failure and hyperthyroidism.

In the present case, patient's age precluded the physiologic causes of gynecomastia (neonatal, puberty-associated or ageing-related). Liver dysfunction was also ruled out by normal Liver Function Test. Hypogonadism was also unlikely as all the secondary sexual characters were normal. Hence, the patient's medications were reviewed and suspected drugs were stopped. If the gynecomastia would not have been reversed after stopping digoxin and furosemide, further investigations could have required exclusion of other causes such as malignancy or thyroid dysfunction, according to the standard algorithm.[5]

The mechanism of digoxin-induced gynecomastia is believed to be a direct action at estrogen receptors due to similarity in the structure of digoxin and estrogen.[6] Although furosemide is reported to cause gynecomastia, its exact mechanism is still not known.

## Conclusion

As digoxin and furosemide are frequently used concurrently, it is important to recognize their possible interaction. Early recognition of the ADR and removal of the offending drug can save the patient from unnecessary investigations and anxiety and also reduce the medical expenses.
